# Voice hearing in young people with posttraumatic stress disorder (PTSD) following multiple trauma exposure

**DOI:** 10.1080/20008066.2024.2435790

**Published:** 2024-12-18

**Authors:** Katie Lofthouse, Ella Beeson, Tim Dalgleish, Andrea Danese, Joanne Hodgekins, Gerwyn Mahoney-Davies, Patrick Smith, Paul Stallard, Jon Wilson, Richard Meiser-Stedman

**Affiliations:** a Department of Clinical Psychology and Psychological Therapies, Norwich Medical School, University of East Anglia, Norwich, UK; b Hertfordshire Partnership University NHS Foundation Trust, St Albans, UK; c MRC Cognition and Brain Sciences Unit, University of Cambridge, Cambridge, UK; d Cambridgeshire and Peterborough NHS Foundation Trust, Cambridge, UK; e Department of Child and Adolescent Psychiatry, King’s College London Institute of Psychiatry, Psychology and Neuroscience, London, UK; f Social, Genetic and Developmental Psychiatry Centre, King's College London, London, UK; g Norfolk and Suffolk NHS Foundation Trust, Norwich, UK; h Cardiff and Vale University Health Board, Cardiff, UK; i Department of Psychology, King’s College London Institute of Psychiatry, Psychology and Neuroscience, London, UK; j Department for Health, University of Bath, Bath, UK

**Keywords:** PTSD, youth, voice hearing, trauma, cognition, panic, TEPT, juventud, escuchar voces, trauma, cognición, pánico, memoria, apreciaciones

## Abstract

**Background:** PTSD is comorbid with a number of other mental health difficulties and the link between voice hearing and PTSD has been explored in adult samples.

**Objective:** To compare the trauma history, symptomatology, and cognitive phenotypes of children and adolescents with a PTSD diagnosis following exposure to multiple traumatic events presenting with voice hearing with those who do not report hearing voices.

**Methods:** Participants (*n* = 120) were aged 8–17 years and had PTSD following exposure to multiple traumas. Three primary analyses were conducted, comparing PTSD symptom severity, prevalence of sexual trauma, and level of negative post-traumatic cognitions between the voice hearing and non-voice hearing groups. Participants were allocated to the voice hearing group if they reported hearing voices in the past two weeks. A range of mental health and cognitive–behavioural factors were considered in exploratory secondary analyses. All analyses were pre-registered.

**Results:** The voice hearing group (*n* = 50, 41.7%) scored higher than the non-voice hearing group (*n* = 70, 58.3%) for negative post-traumatic cognitions, but not PTSD symptom severity or prevalence of sexual trauma. In secondary analyses, the voice hearing group had more sensory-based and fragmented memories and higher scores for panic symptoms than the non-voice hearing group. When participants whose voices were not distinguishable from intrusions or flashbacks were removed from the voice hearing group in a sensitivity analysis, the voice hearing group (*n* = 29, 24.2%) scored higher on negative post-traumatic cognitions and trauma memory quality, with similar effect sizes to the original analysis.

**Conclusions:** Voice hearing is common among youth exposed to multiple traumas with PTSD and is related to cognitive mechanisms proposed to underpin PTSD (appraisals, memory quality) and more panic symptoms. Further research should seek to investigate the underlying mechanisms and directionality for these relationships.

## HIGHLIGHTS

- 41.7% of a sample of young people with PTSD following exposure to multiple traumatic events reported hearing voices.
- The voice hearing group scored higher than the non-voice hearing group on measures of negative post-traumatic cognitions and panic symptoms and had poorer trauma memory quality.
- A sensitivity analysis which excluded participants whose voices were not distinguishable from intrusions or flashbacks replicated the findings for negative post-traumatic cognitions and trauma memory quality.

## Abstract (es)

**Antecedentes:** El TEPT es comórbido con una serie de otras dificultades de salud mental, y el vínculo entre la experiencia de escuchar voces y el TEPT ha sido explorado en muestras de adultos.

**Objetivo:** Comparar la historia de trauma, la sintomatología y los fenotipos cognitivos de niños y adolescentes con diagnóstico de TEPT tras la exposición a múltiples eventos traumáticos que presentan la experiencia de escuchar voces, con aquellos que no reportan escuchar voces.

**Método:** Los participantes (*n* = 120) tenían entre 8 y 17 años, los cuales habían desarrollado TEPT tras la exposición a múltiples traumas. Se realizaron tres análisis principales para comparar la gravedad de los síntomas de TEPT, la prevalencia de trauma sexual y el nivel de cogniciones negativas postraumáticas entre los grupos con y sin experiencia de escuchar voces. Se asignó a los participantes al grupo que escucha voces si declaran haber oído voces al menos en las últimas dos semanas. En los análisis secundarios exploratorios se tuvieron en cuenta diversos factores de salud mental y cognitivo-conductuales. Todos los análisis se registraron previamente.

**Resultados:** El grupo que escucha voces (*n* = 50, 41.7%) obtuvo puntuaciones más altas que el grupo que no escucha voces (*n* = 70, 58.3%) en cogniciones negativas postraumáticas, pero no en la gravedad de los síntomas de TEPT ni en la prevalencia de trauma sexual. En los análisis secundarios, el grupo que escucha voces mostró más recuerdos sensoriales y fragmentados, así como puntuaciones más altas en síntomas de pánico en comparación con el grupo que no escucha voces. Al excluir, en un análisis de sensibilidad, a los participantes cuyas voces no se distinguían de intrusiones o recuerdos retrospectivos (flashbacks), el grupo que escucha voces (*n* = 29, 24.2%) mantuvo puntuaciones más altas en cogniciones negativas postraumáticas y calidad de la memoria del trauma, con tamaño del efecto similar al análisis original.

**Conclusión:** La experiencia de escuchar voces es común entre jóvenes expuestos a múltiples traumas con TEPT y está relacionada con mecanismos cognitivos propuestos como base del TEPT (apreciaciones, calidad de la memoria) y con un mayor número de síntomas de pánico. Se necesitan más investigaciones para examinar los mecanismos subyacentes y la direccionalidad de estas relaciones.

## 1. Background

Post-traumatic stress disorder (PTSD) is a possible reaction to trauma which is associated with a range of poor outcomes related to quality of life and overall functioning, as well as comorbidity with other mental health difficulties in children and adolescents (Lewis et al., 2019). Research into symptoms which are comorbid to PTSD is key for the understanding and effective treatment of young people who have been exposed to trauma. PTSD is frequently comorbid with depression (Angelakis & Nixon, 2015; O’Donnell et al., 2004) and anxiety disorders (Hubbard et al., 1995) but research into comorbid voice hearing is limited. Hearing voices is common in the general population (Watkins, 2008) and can cause significant distress.

Voice hearing is defined by Longden et al. (2012) as ‘a percept-like experience in the absence of appropriate stimulus, which manifests as a human vocalisation, which is experienced in a conscious state and is not induced by organic or state-dependent circumstances’. There are multiple perspectives on how voice hearing is understood in the context of trauma. One is that voice hearing may be considered as a ‘psychotic-like experience’ as it could be an auditory hallucination, classified as a feature of psychotic disorders (DSM-5; American Psychiatric Association, 2013). Psychotic-like experiences are a broad category encompassing ‘subtle, subclinical hallucinations and delusions which are quite common in general population’ (Remberk, 2017).

Psychotic-like experiences are common, reported by approximately 60% of young people (Laurens et al., 2007; Laurens et al., 2012) . Armando et al. (2010) found that psychotic-like experiences in adolescents and young adults (aged 15–26 years) were associated with distress, depression, and poor functioning. When considering research into trauma and psychosis in children, Arseneault et al. (2011) found that children who experienced maltreatment were more likely to report psychotic symptoms at age 12 than children who did not experience maltreatment and Kelleher et al. (2013) observed a bidirectional relationship between childhood trauma and psychosis in a prospective cohort study. Furthermore, Bloomfield et al. (2020) found a relationship between developmental trauma (defined as experiences including emotional, sexual, or physical abuse in childhood or adolescence) and psychosis symptoms during adulthood, which was mediated by dissociation, emotional dysregulation, and PTSD symptoms. However, research investigating the specific link between voice hearing and PTSD has thus far been limited to adult samples; Anketell et al. (2010) found that voice hearing in an adult sample with PTSD diagnosis had a prevalence of 50%.

Alternatively, as voices are experienced as originating outside of oneself, voice hearing can be understood as a dissociative phenomenon. Dissociation is conceptualised as ‘a disruption, interruption, and/or discontinuity of the normal, subjective integration of behaviour, memory, identity, consciousness, emotion, perception, body representation, and motor control’ by the DSM-5 (American Psychiatric Association, 2013), and hearing voices can be understood as one such disruption. Dissociative experiences and psychotic voices may overlap, but they may also be distinguished by features of the voices and comorbid symptoms (e.g. delusions and negative symptoms such as blunted affect are more associated with psychotic voices) (Ross, 2020).

It is important to consider the potential confound between voice hearing and re-experiencing symptoms of PTSD, as intrusions and flashbacks may include experiencing voices related to traumatic events, therefore warranting identification of voice hearing occurring in the absence of re-experiencing symptoms. There could be some overlap between the experiences of intrusions and voice hearing, and there is potential for shared mechanisms such as dissociation and emotional dysregulation (Bloomfield et al., 2020). Hardy et al. (2005) found that in a trauma-exposed adult sample with a diagnosis of nonaffective psychosis, 57.5% of participants had identifiable associations between hallucinations and traumatic experiences, established by assessing the themes and content of the hallucinations in relation to reported traumas. This suggests that there is an overlap between hallucinations and PTSD symptoms, but that it may be possible to distinguish between trauma-related and non-trauma related hallucinations. The present research focused on the experience of voice hearing specifically (rather than the broader category of auditory hallucinations or psychotic-like experiences), because it is a clearly defined and easily measurable construct which may be distressing and clinically meaningful, even in the absence of a psychotic episode or dissociation.

Fundamental questions around the nature of voice hearing in youth with PTSD – including not only its prevalence but also its correlates and potential underlying mechanisms – need to be addressed. Andrew et al. (2008) compared psychiatric and non-psychiatric voice hearers (distinguished by negative and positive appraisals of voice hearing, respectively) and found that current trauma symptoms were a significant predictor of beliefs about voices, suggesting a link between these experiences. In addition, the psychiatric voice hearing group reported a significantly higher level of childhood sexual abuse, with no significant difference between the groups in number of trauma types experienced, which suggests that sexual trauma specifically may be related to the symptom of hearing voices. Furthermore, Anilmis et al. (2015) extended on the importance of appraisals and demonstrated that negative self-beliefs mediate the relationship between the psychological impact of victimisation and psychotic-like experiences in children aged 8–14 years.

Therefore, in light of previous findings, we identified three areas of primary importance to consider. First, given the association between PTSD and a broader range of mental health outcomes (Lewis et al., 2019), the relationship between PTSD severity and voice hearing warrants exploration. Second, trauma *type* (sexual vs non-sexual trauma) may be a key factor in the development of voice hearing. Third, cognitive appraisals warrant consideration, due to the previously identified link between victimisation and psychotic-like experiences in children.

Further to PTSD symptom severity, trauma type, and negative cognitions, there are a range of other psychopathological and cognitive-behavioural factors which could be related to the experience of hearing voices. Psychopathological factors may include complex PTSD (World Health Organization, 2019), dissociation (Longden et al., 2012), depression, anxiety (Lewis et al., 2019), and irritability (Zhang et al., 2022). Cognitive-behavioural factors of interest include trauma memory quality (Meiser-Stedman et al., 2012), safety-seeking behaviours (Alberici et al., 2018), and perceived social support (Daniunaite et al., 2021).

The present study investigated how hearing voices is related to trauma experiences, psychopathological symptom severity, and cognitive-behavioural factors in young people (aged 8–17 years) diagnosed with PTSD following multiple trauma exposure. For the primary analysis, voice hearing and non-voice hearing groups were compared on PTSD symptom severity, sexual trauma prevalence, and negative trauma-related cognitions. Exploratory secondary analyses were conducted comparing these groups on a range of variables covering other psychopathological (i.e. complex PTSD, dissociation, depression, anxiety, and irritability) and cognitive-behavioural (i.e. trauma memory quality, safety-seeking behaviours, and perceived social support) factors. Sensitivity analyses were conducted in which participants whose voices were distinguishable from intrusions were identified and the analyses rerun. In addition, given the importance some commentators have placed on dissociation, we also sought to evaluate this factor as a confounding variable.

Our primary hypotheses were that the voice hearing group would have significantly higher scores than the non-voice hearing group on PTSD symptom severity and negative cognitions and a significantly higher rate of sexual trauma. These aimed to replicate associations established by previous research: that voice hearing is associated with greater PTSD symptom severity, heightened negative cognitions, and experience of sexual trauma. Our secondary exploratory hypothesis was that the voice hearing group would have significantly higher scores than the non-voice hearing groups on other measures of psychopathological and cognitive-behavioural factors.

## 2. Methods

### 2.1. Design

The present study was a cross-sectional design comprising of analysis of the baseline data from the Delivery of Cognitive Therapy for Young People after Trauma (DECRYPT) trial (Allen et al., 2021), a randomised controlled trial of cognitive therapy for PTSD in youth exposed to multiple traumatic stressors. Measures were selected from the battery of self-report and parent/caregiver-report interviews and questionnaires to assess PTSD symptom severity, prevalence of sexual trauma, and negative post-traumatic cognitions for the primary analysis. For the secondary analysis, measures assessing dissociation, depression, anxiety, irritability, trauma memory quality, safety behaviours, and social support were identified. These analyses were pre-registered on the Open Science Framework (https://osf.io/q85rz/).

### 2.2. Ethical considerations

Ethical approval for the DECRYPT trial was provided by UK Health Research Authority Research Ethics Committee (East of England – Cambridge South, 16/EE/0233). For participants aged under 16 years, informed consent was provided by parents and caregivers, and the child or young person was also asked to give their assent. Participants aged 16 years or older could provide informed consent without their parent or caregiver.

### 2.3. Participants

The sample size of 120 participants was determined by the primary outcome of the DECRYPT trial (Allen et al., 2021). Participants were drawn from Child and Adolescent Mental Health Services (CAMHS) and Youth Services in Cambridgeshire, Cardiff, Essex, Hertfordshire, Kent, Norfolk, South London, and Suffolk. Inclusion criteria required participants to be aged 8–17 years with a diagnosis of PTSD (as defined by DSM-5 and diagnosed using the CPSS-I-5, Child PTSD Symptom Scale – Interview version, Foa et al. [2018]) following multiple trauma exposure, and to have a score equal to or greater than 17 on the Child Revised Impact of Events Scale, 8-item version (Perrin et al., 2005). All participants also met ICD-11 criteria for PTSD. Exclusion criteria were a change of prescribed psychiatric medication within the past two months, PTSD symptoms relating exclusively to one trauma, pervasive developmental or neurodevelopmental disorder, intellectual disability, another primary psychiatric diagnosis or clinical need warranting treatment ahead of PTSD (e.g. psychosis), inability to speak English, ongoing exposure to threat, strong likelihood of being unable to complete treatment (e.g. imminent house move), or history of organic brain damage. Table 1 contains the demographic and trauma history data for the sample.

**Table 1.** Sample demographic characteristics.

	Whole sample *n* = 120	Voice hearing sample (*n *= 50)	Non-voice hearing sample (*n* = 70)
Age in years, mean (*SD*)	14.9 (2.5)	15.1 (2.3)	14.8 (2.7)
Gender, *n* (%)			
Male	33 (27.5)	12 (24.0)	21 (30.0)
Female	87 (72.5)	38 (76.0)	49 (70.0)
Ethnicity, *n* (%)			
White (any background)	96 (80.0)	42 (84.0)	55 (78.5)
Black (any background)	9 (7.5)	1 (2.0)	8 (11.4)
Asian (any background)	2 (1.7)	1 (2.0)	1 (1.4)
Mixed (any background)	11 (9.2)	5 (10.0)	6 (8.6)
Any other ethnic group	1 (0.8)	1 (2.0)	0 (0.0)
Ethnicity not stated	1 (0.8)	0 (0.0)	0 (0.0)
Traumatic Experiences, *n* (%)			
Natural disaster	3 (2.5)	2 (4.0)	1 (1.4)
Accident	34 (28.3)	17 (34.0)	17 (24.3)
Robbed	10 (8.3)	5 (10.0)	5 (7.1)
Physical abuse inside family	57 (47.5)	25 (50.0)	32 (45.7)
Physical abuse outside family	57 (47.5)	24 (48.0)	33 (47.1)
Witnessed physical abuse inside family	66 (55)	32 (64.0)	34 (48.6)
Witnessed physical abuse outside family	53 (44.2)	24 (48.0)	29 (41.4)
Inappropriate sexual contact	36 (30)	19 (38.0)	17 (24.3)
Someone forcing/pressuring sex	30 (25)	15 (30.0)	15 (21.4)
Sudden death/injury of a close person	55 (45.8)	23 (46.0)	32 (45.7)
Attacked, stabbed, shot at, or hurt badly	13 (10.8)	7 (14.0)	6 (8.6)
Witnessed someone attacked, stabbed, shot at, or hurt badly	35 (29.2)	15 (30.0)	20 (28.6)
Medical procedure	29 (24.2)	17 (34.0)	12 (17.1)
Exposure to war	0 (0.0)	0 (0.0)	1 (1.4)
Other	83 (69.2)	32 (64.0)	51 (72.9)
Number of Trauma Types, mean (SD)	4.7 (2.2)	5.1 (2.2)	4.3 (2.11)

### 2.4. Measures

#### 2.4.1. Voice hearing interview

The voice hearing interview is a child-report structured interview comprised of six items, four of which were taken from the Unusual Experiences Questionnaire (UEQ) (Anilmis et al., 2015; Laurens et al., 2012). The UEQ has good internal consistency (Laurens et al., 2012). The question establishing the presence of voices (‘Have you ever heard voices that other people could not hear?’) was measured on a three-point Likert-type scale from ‘Not true’ to ‘Certainly true’. The other three items concerning the frequency, distress, and impairment related to hearing voices were assessed on a four-point Likert-type scale from ‘Not at all’ to ‘A great deal’. Two additional items were included to explore how the voices relate to trauma and PTSD symptoms (‘Were these voices of the people that attacked you?’ and ‘Were these voices part of your intrusive thoughts or flashbacks?’). These were measured on a three-point Likert-type scale from ‘Not true’ to ‘Certainly true’. In addition, interviewers completed an open response item clarifying the nature and content of voices.

Participants were included in the voice hearing group if they endorsed hearing at least one voice in the preceding two weeks. A sensitivity analysis was conducted whereby information from the two items linking voices to PTSD symptoms and the open response item regarding the content of voices was analysed to exclude participants from the voice hearing group whose voices appeared to be exclusively flashback or intrusion related, or any participants with insufficient information to establish this. Two authors (KL and RMS) independently reviewed the voice content open response item and disagreements were discussed at a consensus meeting to reach full agreement.

#### 2.4.2. Children’s revised impact of event scale, 8-item version (CRIES-8)

The CRIES-8 (Perrin et al., 2005) is a self-report questionnaire measure assessing frequency of post-traumatic stress symptoms over the preceding seven days. It has good face, construct, predictive, and criterion validity (Perrin et al., 2005; Stallard et al., 1999), *α* = .66 in this sample.

#### 2.4.3. Child and adolescent trauma screen (CATS)

The CATS (Sachser et al., 2017) has self-report and caregiver-report versions, both of which were employed in the present study as a structured interview. For the present research, the first 15 items pertaining to trauma history were analysed; these list 14 different trauma types and an open answer question to accommodate any non-listed trauma types, with the participant asked to indicate if they have experienced each event as a yes or no question; caregivers were asked the same with regards to the young person in their care. One of the participant or their parent/caregiver needed to endorse a sexual trauma for the participant to meet the sexual trauma criterion.

#### 2.4.4. Post-Traumatic cognitions inventory – child version (CPTCI)

The CPTCI (Meiser-Stedman et al., 2009) is a 25-item self-report questionnaire assessing negative appraisals over the preceding two weeks of one or more of a participant’s traumatic experiences. The scale comprises two subscales, a sense of ‘permanent and disturbing change’ and a sense of being a ‘fragile person in a scary world’. The measure has good internal consistency, test-retest reliability, convergent validity, and discriminative validity (Meiser-Stedman et al., 2009), α = .94 in this sample.

#### 2.4.5. Complex PTSD interview

To establish whether participants met the criteria for complex PTSD, as defined by ICD-11 (World Health Organization, 2019), a three-item self-report structured diagnostic interview was conducted. The interview was devised by the DECRYPT trial team (see supplementary material) based on ICD-11 draft criteria (World Health Organization, 2019). The three interview items correspond to the three disturbances in self-organisation (DSO) symptoms defined in ICD-11: affective dysregulation, negative self-concept, and difficulties in sustaining relationships. Each item had one introductory question assessing the overall symptom, with optional follow-up questions for positive responses. Each of the three DSO symptoms was assessed on a five-point Likert-type scale from zero (‘Not at all’) to four (‘Six or more times a week/almost always’), consistent with the CPSS-I-5.

#### 2.4.6. Revised child anxiety and depression scale (RCADS)

The RCADS (Chorpita et al., 2000) is a 47-item self-report questionnaire assessing symptoms in the preceding two weeks corresponding to anxiety disorders and depression in young people. The measure has good internal consistency (Kösters et al., 2015), test-retest reliability, and convergent validity (Chorpita et al., 2000), *α* = .94 in this sample.

#### 2.4.7. Dissociation

Dissociation was measured using a three-item questionnaire assessing symptoms experienced during the preceding two weeks. Items were scored on a four-point Likert-type scale from ‘Not at all or only one time’ to ‘Five or more times a week/almost always’, *α* = .73 in this sample.

#### 2.4.8. Strengths and difficulties questionnaire (SDQ)

The SDQ (Goodman, 1997) is a 25-item caregiver-report measure assessing emotional symptoms, conduct problems, hyperactivity/inattention, peer relationship problems, and prosocial behaviour, with each scale comprised of five items. The first four scales (20 items), excluding prosocial behaviour, are used to calculate a total difficulties score, used in the present research. The total difficulties score has acceptable test-retest reliability (Bergström & Baviskar, 2021), and sufficient convergent, discriminant, and criterion validity (Vugteveen et al., 2021), *α* = .66 in this sample.

#### 2.4.9. Affective reactivity index – child version (ARI-C)

The ARI-C (Stringaris et al., 2012) is a seven-item self-report measure of irritability which asks participants to rate irritability symptoms compared to others of the same age (e.g. ‘I am easily annoyed by others’), *α* = .94 in the sample.

#### 2.4.10. Trauma memory quality questionnaire (TMQQ)

The TMQQ (Meiser-Stedman et al., 2007) is an 11-item self-report questionnaire which assesses the current characteristics of trauma memories; particularly the extent to which they are composed of sensory elements. The measure has good internal consistency, criterion validity, and convergent validity (Meiser-Stedman et al., 2007). Higher scores indicate more sensory-based and fragmented memories, *α* = .73 in this sample.

#### 2.4.11. Child safety behaviour scale (CSBS)

The CSBS (Alberici et al., 2018) is a 13-item self-report questionnaire assessing safety behaviours (strategies employed to prevent a dreaded outcome, Salkovskis et al., 1999) over the past two weeks. The measure has excellent internal consistency and good discriminant validity and specificity (Alberici et al., 2018), α = .81 in this sample.

#### 2.4.12. Multidimensional scale of perceived social support (MSPSS)

The MSPSS (Zimet et al., 1988) is a 12-item self-report questionnaire measuring a participant’s perceptions of support from family, friends, and a significant other. The measure has good internal reliability (Zimet et al., 1988) and good convergent and discriminative validity (De Maria et al., 2018), *α* = .82 in this sample.

### 2.5. Data analysis

The sample size was predetermined by the DECRYPT trial. A power analysis conducted using G*Power version 3.1.9.7 (Faul et al., 2007) indicated that two groups (*n* = 50 and *n* = 70) with a significance criterion of *α* = .05 for a test of means comparisons would have 80% power to detect an effect size (standardised mean difference) of .52. Statistical analysis was conducted using IBM SPSS Statistics Version 28 (IBM Corp. 2021). Data were assessed for assumptions of normality, skewness, and kurtosis (see Supplementary Material). The scores for the CRIES-8, SDQ, ARI-C, total RCADS, and Dissociation did not meet the normality assumption. For the CRIES-8, ARI-C, and Dissociation scores, no adequate transformations could be found; therefore, non-parametric Mann-Whitney tests were conducted for these variables. The scores for the SDQ and total RCADS met the normality assumption after a square root transformation, allowing parametric tests to be conducted as planned. Independent samples t-tests were conducted to compare the voice hearing and non-voice hearing groups on scores for the following variables: CRIES-8, CPTCI, RCADS total and subscales (depression, panic, generalised anxiety disorder), SDQ, TMQQ, CSBS-13, and MSPSS. Prevalence of sexual trauma and complex PTSD diagnosis were compared between the voice hearing and non-voice hearing groups using chi-square tests due to the categorical nature of these variables. Corrections were applied to adjust for multiple comparisons. For the primary analysis (CRIES-8 score, sexual trauma prevalence, and CPTCI score), a Bonferroni correction was applied. For the exploratory secondary analysis comprising all other variables, a Holm-Bonferroni correction was applied.

Levene’s test for equal variances was conducted for all t-tests; this was not significant and equal variances were assumed unless otherwise specified. Cohen’s d effect sizes were calculated.

## 3. Results

### 3.1. Descriptive statistics

The sample comprised 120 participants, mean age 14.9 years (*SD* 2.5 years), 72.5% female, 96% white. Table 2 contains descriptive statistics of all measures. Fifty of 120 participants (41.6%) reported hearing voices in the preceding two weeks. Table 3 contains statistics regarding the characteristics of voices.

**Table 2.** Descriptive statistics for all measures.

Measure	*n*	*M*	*SD*	Range	Cronbach’s *α*
PTSD symptoms (CRIES-8)	120	31.6	6.00	17–40	.66
Negative cognitions (CPTCI)	118	73.6	15.9	26–100	.94
RCADS total score	120	82.1	24.8	20–133	.94
Anxiety (RCADS)	120	12.0	3.93	2–18	.78
Depression (RCADS)	120	19.5	6.24	2–30	.83
Panic disorder (RCADS)	120	14.4	7.12	0–27	.91
Parent-rated emotional difficulties (SDQ)	94	21.5	6.13	9–35	.66
Irritability (ARI-C)	120	7.93	4.21	0–14	.94
Memory quality (TMQQ)	120	31.8	5.50	17–44	.73
Safety behaviours (CSBS)	114	35.7	7.30	15–51	.81
Social support (MSPSS)	119	57.8	13.4	17–84	.82
Dissociation	120	6.87	2.50	3–12	.73
Sexual Trauma*	50				
Complex PTSD Diagnosis*	72				

Note: CRIES-8 = Child Revised Impact of Events Scale; CPTCI – Post-Traumatic Cognitions Inventory, Child version; RCADS = Revised Child Anxiety and Depression Scale; SDQ = Strengths and Difficulties Questionnaire; ARI-C = Affective Reactivity Index – Child version; TMQQ = Trauma Memory Quality Questionnaire; CSBS = Child Safety Behaviour Scale; MSPSS = Multidimensional Scale of Perceived Social Support.

* Sexual trauma and Complex PTSD diagnosis are categorical variables, so the frequency of each of these within the sample is reported here.

**Table 3.** Frequencies for characteristics of voices.

Question	***n (%)***			
	*Not at all*	*Only a little*	*Quite a lot*	A great deal
How much has it upset you?	4 (8%)	11 (22%)	22 (44%)	13 (26%)
How hard has it made things at home or school?	7 (14%)	9 (18%)	20 (40%)	14 (28%)

Question	***n (%)***			
	*Not true*	*Somewhat true*	*Certainly true*	
Were these the voices of the people that attacked you?	24 (48%)	9 (18%)	17 (34%)	
Were these voices part of your intrusive thoughts or flashbacks?	14 (28%)	20 (40%)	15 (30%)	

### 3.2. Demographic analyses

There was no difference between the voice hearing and the non-voice hearing groups on mean age, mean number of trauma types, proportion of female participants, or proportion of non-white participants.

### 3.3. Primary analyses

Table 4 contains mean scores differentiated by group. With respect to PTSD symptom severity, a Mann-Whitney test indicated that despite the voice hearing group having a higher mean score, there was no significant difference between the voice hearing group and the non-voice hearing group (*p *= .046; Bonferroni correction required *p* = . 0167; Cohen’s *d* = .37). There was no relationship between hearing voices and sexual trauma (*p *= .18, Cohen’s *d* = .28). With respect to negative trauma-related cognitions, an independent samples t-test was conducted. The voice hearing group scored higher than the non-voice hearing group (*p* = .014; Cohen’s *d* = .45).

**Table 4.** Between groups analysis for primary and secondary outcomes.

Measure	Voices group *(n = 50), m (SD)*	No voices group *(n = 70), m (SD)*	Test statistic	*p*	Effect size (*Cohen’s d*)
Primary analysis					
** Negative cognitions (CPTCI)**	**77.7** (**13.1)**	**70.7** (**17.2)**	***t* = 2.49**	.**014**	.**445**
** **PTSD symptoms (CRIES-8)	32.9 (5.07)	30.6 (6.42)	*U* = 1377.0	.046	.366
** **Sexual trauma*	24 (48%)	25 (35.7%)	*χ*^2 ^= 1.82	.177	.280
Secondary Analysis					
** Memory quality (TMQQ)**	**34.2** (**4.94)**	**30.0** (**5.25)**	***t* = 4.36**	**<**.**001**	.**807**
** Panic disorder (RCADS)**	**16.6** (**6.20)**	**12.9** (**7.36)**	***t* = 2.90**	.**004**	.**537**
** **RCADS total score	88.7 (22.9)	77.4 (25.2)	*t* = 2.54	.012	.470
** **Anxiety (RCADS)	12.9 (3.61)	11.3 (4.05)	*t* = 2.12	.036	.392
** **Safety behaviours (CSBS)	24.3 (6.60)	21.4 (7.60)	*t* = 2.10	.038	.397
** **Dissociation	7.36 (2.39)	6.51 (2.53)	*U* = 1391.5	.055	.355
** **Parent-rated emotional difficulties (SDQ)	22.8 (6.08)	20.6 (6.04)	*t* = 1.77	.080	.369
** **Depression (RCADS)	20.4 (6.23)	18.9 (6.21)	*t* = 1.35	.181	.249
** **Irritability (ARI-C)	8.73 (4.43)	7.36 (3.98)	*U* = 1389.5	.054	.357
** **Complex PTSD diagnosis*	31 (62%)	41 (58.6%)	*χ*^2 ^= 0.143	.705	.013*
** **Social support (MSPSS)	57.7 (14.0)	57.9 (13.0)	*t* = 0.068	.946	.013

Note: Significant results depicted in bold. A Bonferroni correction was applied for the three primary analyses and a Holm-Bonferroni correction was applied for the secondary analysis.

CPTCI – Post-Traumatic Cognitions Inventory, Child version; CRIES-8 = Child Revised Impact of Events Scale; TMQQ = Trauma Memory Quality Questionnaire; RCADS = Revised Child Anxiety and Depression Scale; CSBS = Child Safety Behaviour Scale; SDQ = Strengths and Difficulties Questionnaire; ARI-C = Affective Reactivity Index – Child version; MSPSS = Multidimensional Scale of Perceived Social Support.

*Categorical variables so frequencies rather than means are reported.

### 3.4. Secondary analyses

Differences were found between voice hearing and non-voice hearing on measures of trauma memory quality and panic disorder. The voice hearing group had a higher TMQQ score than the non-voice hearing group, indicating more sensory-based and poorly verbalised memories, with a large effect size, Cohen’s *d* = .81. The voice hearing group had a higher score on the RCADS panic disorder subscale than the non-voice hearing group (Cohen’s *d* = .54). There were no differences between the voice hearing and non-voice hearing groups on the other secondary measures.

### 3.5. Sensitivity analyses

A sensitivity analysis was conducted in which participants whose voices did not appear to be distinguishable from intrusions or flashbacks, or with insufficient information to conclude this, were excluded from the analysis, resulting in a group of 29 participants in the voice hearing group and 70 in the non-voice hearing group (see Supplementary Material).

The full sensitivity analysis is reported in the Supplementary Material. Negative trauma-related cognitions remained higher for the voice hearing group (*p* = .01, Cohen’s *d* = .59). Trauma memory quality remained (*p *< .001, Cohen’s *d* = .82), but there was no longer a difference between the groups on panic disorder symptoms (*p *= .025, Cohen’s *d* = .50). There was no difference between the sensitivity analysis groups on demographic factors.

Further analyses were conducted to consider the potential confounding effect of dissociation. Logistic regression models were determined where voice hearing status was the dependent variable and the variables identified as significant (negative post-traumatic cognitions, trauma memory quality, and panic disorder) were entered as independent variables alongside dissociation score (see Supplementary Materials Tables S3 and S4). The results for trauma memory quality and panic disorder remained, but negative trauma-related cognitions were not a predictor of voice hearing after controlling for dissociation.

## 4. Discussion

The present study is a novel investigation of the experience of voice hearing in children and adolescents with a PTSD diagnosis following multiple trauma exposure, with comparisons made between the voice hearing and non-voice hearing groups on trauma type and psychopathological and cognitive–behavioural factors. As hypothesised, the voice hearing group scored higher on a measure of negative cognitions than the non-voice hearing group. There was no evidence for a difference in PTSD symptom severity or incidence of sexual trauma between the groups, contrary to our other primary hypotheses. From the secondary analyses, the voice hearing group had worse trauma memory quality (more fragmented, sensory based, poorly verbalised memories) and more severe panic symptoms than the non-voice hearing group, partially endorsing the secondary hypothesis that the voice hearing group would score higher on measures of psychopathology and cognitive–behavioural factors. However, no differences were found between the groups on incidence of complex PTSD diagnosis or on measures of dissociation, depression, generalised anxiety disorder, irritability, safety behaviours, or perceived social support.

The first finding of note was the prevalence of voice hearing. Fifty of 120 participants (41.7%) endorsed at least one incidence of voice hearing in the preceding two weeks. Furthermore, most voice hearers were at least ‘quite a lot’ upset by their experience of voice hearing. Disentangling the experience of voice hearing from trauma-related themes or PTSD symptoms was difficult, with just over half of the voice hearing group reporting that the voices they heard were those of their attackers. However, a significant proportion of voice hearers (34%) confirmed that the voices they heard did *not* form part of their re-living symptoms. This is comparable to previous research in which 27% of young people aged 15–25 years presenting with post-traumatic intrusions and hallucinations following a first episode of psychosis experienced hallucinations which were not related to their post-traumatic intrusions (Peach et al., 2021).

There are a range of models relevant to understanding the link between PTSD and voice hearing. Hardy (2017) proposed a trauma-informed model of voices suggesting that trauma increases the risk of unhelpful emotion regulation, distorted trauma memories, and alterations to appraisals. These three vulnerability factors can lead to trauma memory intrusions (re-experiencing symptoms of PTSD) and anomalous experiences such as voice hearing. This model proposes that PTSD intrusions and hallucinations lie on a continuum of memory fragmentation following trauma. The present findings support this given the results for poorer trauma memory quality and negative post-traumatic appraisals.

Between-groups comparisons found that the voice hearing group scored higher on a measure of negative cognitions than the non-voice hearing group. The importance of negative schematic beliefs in adolescents was demonstrated by Anilmis et al. (2015), who found that negative beliefs about the self and others mediated the relationship between bullying and distressing unusual experiences in a sample aged 8–14 years. The importance of negative cognitions aligns with the cognitive model of PTSD (Ehlers & Clark, 2000), in which negative appraisals can contribute to a sense of current threat, and also with the cognitive model of psychosis (Garety et al., 2001), in which negative cognitions mediate the relationship between negative experiences and positive symptoms of psychosis. The relationship between voice hearing and negative cognitions held in the sensitivity analysis when focusing only on non-flashback voices, supporting the cognitive model of psychosis as a potential mechanism for voice hearing experiences. However, this relationship was no longer significant when controlling for symptoms of dissociation, suggesting the experience of voice hearing may be generated through dissociative mechanisms. A further possibility is that experiences of voice hearing could result in more negative appraisals regarding a sense of being damaged or vulnerable, as the present research does not provide information regarding the directionality of effects. From the secondary analyses, the voice hearing group had more sensory-based, poorly verbalised trauma memories. The effect for trauma memory was noteworthy in its size (*d* > .8), and its persistence in sensitivity analyses. These findings support that more fragmented memories may play a role in voice hearing for trauma-exposed youth. In the Ehlers and Clark (2000) cognitive model of PTSD, poor memory quality contributes to a sense of current threat, which may then increase the risk of voice hearing. Similarly, in the Hardy (2017) model, distorted trauma memories are linked to greater vulnerability to anomalous experiences such as voice hearing.

A further finding was that the voice hearing group experienced more severe panic symptoms than the non-voice hearing group. The negative appraisals underpinning PTSD may overlap with the catastrophic misinterpretations involved in the development of panic (Clark, 1986), which could worsen a sense of threat and thus lead to heightened panic symptoms in those hearing voices. A further common mechanism in the cognitive model may be greater attention towards bodily and cognitive phenomena. The Hardy (2017) model also suggests that exposure to trauma increases the risk of emotion dysregulation, which panic symptoms may be indicative of. Alternatively, voice hearing could pre-date trauma and may increase the frequency of panic episodes and the likelihood of negative appraisals. The lack of evidence exhibiting a difference between the voice hearing and non-voice hearing groups on the other psychopathological and cognitive factors included in the secondary analyses is also noteworthy, but this may be attributed to lack of power to detect small effects.

The present research demonstrates that voice hearing within youth with PTSD following multiple trauma exposure is a common and distressing experience, so could have clinical implications as a treatment target. Screening for voice hearing, associated distress, and characteristics of voices may inform treatment for PTSD. Maddox et al. (2013) demonstrated that CBT for unusual (psychotic-like) experiences in children is effective, so incorporating these techniques into cognitive therapy for PTSD to address voice hearing may enhance treatment efficacy for young people presenting with voice hearing. Nevertheless, the present study suggests that targeting the cognitive pathways proposed in the Ehlers and Clark (2000) model of PTSD (in particular appraisals and trauma memory quality) may help to reduce voice hearing symptoms through mechanisms common to both PTSD and voice hearing. Longitudinal analysis of participants in the DECRYPT trial could compare the response of voice hearing and non-voice hearing participants to trauma-focused CBT.

The strengths and limitations of the present study should be noted. The study design was robust, with a sample comprised of young people with a PTSD diagnosis after exposure to multiple traumatic events, resulting in a powerful control group to compare with but also reducing generalisability to non-PTSD or non-trauma samples. To ensure methodological rigour, the study design and hypotheses were pre-registered and a correction for multiple comparisons was used. The robustness of the results was also confirmed using sensitivity analyses. The gender distribution was skewed towards females, but this is reflective of wider PTSD research (Meiser-Stedman et al., 2017; Sachser & Goldbeck, 2016). As the sample size was predetermined by the DECRYPT trial, the power afforded, whilst adequate, was only able to detect medium-sized effects. The dissociation questionnaire and complex PTSD interview have not previously been validated and some questionnaires (in particular the CRIES-8 and the SDQ) produced low scores for reliability. In addition, the dissociation questionnaire used was brief, with items selected to reflect DSM-5 depersonalisation and derealisation, and therefore covered a narrower range of dissociative symptoms relative to other measures of dissociation such as the Dissociative Experiences Scale (Bernstein et al., 1986), which also contains items related to dissociative identity. Similarly, a measure of psychosis would have strengthened the research by confirming whether participants experienced other symptoms consistent with psychosis beyond just voice hearing.

Several areas could be researched further to better understand the relationship between voice hearing and the identified psychopathological (panic symptoms) and cognitive (negative cognitions and trauma memory quality) factors. As the current findings involved a cross-sectional design, investigation of the underlying mechanisms relating negative cognitions, trauma memory quality, and panic symptoms to voice hearing may be clarified through a longitudinal design to determine the direction of these relationships and whether they are interconnected. Similar research with a single-trauma PTSD group or a non-trauma group could be useful to investigate how experience or frequency of trauma may relate to voice hearing. In addition, the relationship between, and overlap of, voice hearing and flashback symptoms warrants investigation, given that these were distinct experiences for some participants but not for others. Furthermore, research distinguishing between psychotic and dissociative experiences of voice hearing may be useful for elucidating the underlying mechanisms.

In conclusion, this study has demonstrated that voice hearing occurs in a significant proportion of young people with a PTSD diagnosis following exposure to multiple traumatic events, and that voice hearing and non-voice hearing groups differ with regards to negative cognitions, trauma memory quality, and panic symptoms. Future research should explore how these factors are related and investigate management of voice hearing in treatment of trauma-exposed youth.

## Supplementary Material

Supplementary Material.docx

## Data Availability

As this research used data from a randomised controlled trial, data will be made available after publication of the main trial paper for participants who consented to sharing their data.
