# Supplementary material for: Voice hearing in young people with posttraumatic stress disorder (PTSD) following multiple trauma exposure
Source: Eur J Psychotraumatol. 2024 Dec 18;15(1):2435790. doi: 10.1080/20008066.2024.2435790 (PMC11656750; doi:10.1080/20008066.2024.2435790)
Supplement: Supplementary Material.docx [file ZEPT_A_2435790_SM0052.docx]

**Supplementary Material**

***Complex PTSD Interview***

**COMPLEX ITEMS**

“Following onset of the stressor event and co-occurring with PTSD symptoms, there is the development of persistent and pervasive impairments in affective, self and relational functioning including problems in affect regulation, persistent beliefs about oneself as diminished, defeated or worthless, persistent difficulties in sustaining relationships” [ICD-11 Working Group, January 31, 2012]

Problems in affect dysregulation are characterized by heightened emotional reactivity to and difficulty recovering from minor stressors, violent outbursts, reckless or self-destructive behaviour and tendency towards dissociative states when under stress. In addition, there may be emotional numbing, particularly a lack of ability to experience pleasure or positive emotions.

The individuals also develops persistent beliefs about himself or herself as diminished, defeated or worthless accompanied by deep and pervasive feelings of shame, guilt or failure related to, for example, not having escaped from or succumbing to the adverse circumstance, or not having been able to prevent the suffering of others.

There are also persistent difficulties in sustaining relationships. This may present in a variety of ways and is characterized primarily by difficulties in feeling close to others. The person may consistently avoid, deride or have little interest in relationships and social engagement more generally. Alternatively there may be occasional intense relationships but the person has difficulty sustaining them.

| **0** | | **1** | **2** | **3** | | | **4** | | | |
| --- | --- | --- | --- | --- | --- | --- | --- | --- | --- | --- |
| **Not at all** | | **Once a week or less/a little** | **2 to 3 times a week/somewhat** | **4 to 5 times a week/a lot** | | | **6 or more times a week/almost always** | | | |
| 1. | *AFFECTIVE DYSREGULATION*  Do you get really upset by things that don’t upset other people?  *Follow-up questions for positive responses*:  How often in the past month have you got really upset by small things?  When you get upset do you find it hard to control your temper?  When you get upset about things, how long does it take you to calm down?  Do your feelings tend to be easily hurt?  Do you find it hard to enjoy things or feel positive feelings? | | | | 0 | 1 | | 2 | 3 | 4 |
| 2. | *NEGATIVE SELF-CONCEPT*  Do you feel like you are no good?  *Follow-up questions for positive responses*:  How often do you feel like you’re no good?  Do you feel like you don’t matter or that you’re worthless?  How strong is this feeling?  Do you feel guilty or ashamed of yourself? | | | | 0 | 1 | | 2 | 3 | 4 |
| 3. | *DIFFICULTIES IN SUSTAINING RELATIONSHIPS*  Do you find it difficult to have good friends?  *Follow-up questions for positive responses*:  Do you find it easier not to have friends?  Have you given up on having friends?  Do you find friendships are always difficult and leave you feeling hurt?  How interested are you in getting closer friends? | | | | 0 | 1 | | 2 | 3 | 4 |

***Dissociation Interview***

**DISSOCIATION (post-event)**

Specify whether:

**With dissociative symptoms**: The individual's symptoms meet the criteria for post-traumatic stress disorder, and in addition, in response to the stressor, the individual experiences persistent or recurrent symptoms of either of the following:

1. **Depersonalization**: Persistent or recurrent experiences of feeling detached and as if one were an outside observer of, one's mental processes or body (e.g. feeling as though one were in a dream; feeling a sense of unreality of self or body or of time moving slowly).

2. **Derealization**: Persistent or recurrent experiences of unreality of surroundings (e.g., the world around the individual is experienced as unreal, dreamlike, distant or distorted).

**Note**: To use this subtype, the dissociative symptoms must not be attributable to the physiological effects of a substance (e.g., blackouts, behavior during alcohol intoxication) or another medical condition (e.g., complex partial seizures).

| **0** | | **1** | **2** | **3** | | | **4** | | | |
| --- | --- | --- | --- | --- | --- | --- | --- | --- | --- | --- |
| **Not at all** | | **Once a week or less/a little** | **2 to 3 times a week/somewhat** | **4 to 5 times a week/a lot** | | | **6 or more times a week/almost always** | | | |
| 1. | **Depersonalization**  Have you felt as if you were outside your body?  *Follow-up questions for positive responses*:  Have you felt as if your body doesn’t really belong to you?  Has it felt like time is moving very slowly?  Have you felt as if you’re not really where you actually are?  How often has this happened in the past month?  How strong is this feeling?  *CONFIRM THAT NOT RELATED TO ALCOHOL OR OTHER SUBSTANCES* | | | | 0 | 1 | | 2 | 3 | 4 |
| 2. | **Derealization**  Have you felt as if things around you weren’t real?  *Follow-up questions for positive responses*:  Have you felt as if you were in a dream or a film/movie?  Do things around you seem strange or unusual?  How often has this happened in the past month?  How strong is this feeling?  *CONFIRM THAT NOT RELATED TO ALCOHOL OR OTHER SUBSTANCES* | | | | 0 | 1 | | 2 | 3 | 4 |

***Table S1: Normality Test***

| Measure | Kolmogorov-Smirnov | |
| --- | --- | --- |
|  | Statistic | *p* |
| Negative cognitions (CPTCI) | .088 | .086 |
| **PTSD symptoms (CRIES-8)** | **.162** | **<.001** |
| Memory quality (TMQQ) | .063 | .200 |
| Panic disorder (RCADS) | .094 | .052 |
| **RCADS total score** | **.098** | **.033** |
| Anxiety (RCADS) | .088 | .088 |
| Safety behaviours (CSBS) | .089 | .076 |
| **Dissociation** | **.107** | **.014** |
| **Parent-rated emotional difficulties (SDQ)** | **.099** | **.030** |
| Depression (RCADS) | .080 | .200 |
| **Irritability (ARI-C)** | **.120** | **.003** |
| Social support (MSPSS) | .069 | .200 |
| Transformed Variables |  |  |
| **PTSD symptoms (CRIES-8) square root** | **.175** | **<.001** |
| RCADS total score square root | .078 | .200 |
| **Dissociation square root** | **.129** | **<.001** |
| Parent-rated emotional difficulties (SDQ) square root | .071 | .200 |
| **Irritability (ARI-C) square root** | **.121** | **.003** |

**Note:** Significant results depicted in bold; a significant result indicates that the distribution of that variable is significantly different from a normal distribution.
CPTCI – Post-Traumatic Cognitions Inventory, Child version; CRIES-8 = Child Revised Impact of Events Scale; TMQQ = Trauma Memory Quality Questionnaire; RCADS = Revised Child Anxiety and Depression Scale; CSBS = Child Safety Behaviour Scale; SDQ = Strengths and Difficulties Questionnaire; ARI-C = Affective Reactivity Index – Child version; MSPSS = Multidimensional Scale of Perceived Social Support.

***Table S2: Sensitivity Analysis (Participants with voices indistinguishable from flashbacks/intrusions removed)***

| Measure | Voices group *(n=29), m (SD)* | No voices group *(n=70), m (SD)* | Test Statistic | *p* | Effect Size (*Cohen’s d*) |
| --- | --- | --- | --- | --- | --- |
| Primary Analysis |  |  |  |  |  |
| **Negative cognitions (CPTCI)** | **80.3 (13.2)** | **70.7 (17.2)** | ***t* = 1.80** | **.01** | **.588** |
| PTSD symptoms (CRIES-8) | 33.7 (4.83) | 30.6 (6.42) | *U* = 723.5 | .024 | .466 |
| Sexual trauma* | 14 (48.3%) | 25 (35.7%) | *χ^2^* = 1.36 | .244 | .324 |
| Secondary Analysis |  |  |  |  |  |
| **Memory quality (TMQQ)** | **34.3 (5.00)** | **30.0 (5.25)** | ***t* = 3.73** | **<.001** | **.823** |
| Panic disorder (RCADS) | 16.4 (6.59) | 12.9 (7.36) | *t* = 2.28 | .025 | .503 |
| RCADS total score | 88.4 (24.1) | 77.4 (25.2) | *t* = 1.97 | .052 | .435 |
| Anxiety (RCADS) | 12.8 (3.89) | 11.3 (4.05) | *t* = 1.60 | .112 | .354 |
| Safety behaviours (CSBS) | 38.1 (6.96) | 34.4 (7.60) | *t* = 2.23 | .028 | .498 |
| Dissociation | 7.69 (2.59) | 6.51 (2.53) | *U* = 746.5 | .038 | .427 |
| Parent-rated emotional difficulties (SDQ) | 23.3 (7.26) | 20.6 (6.04) | *t* = 1.58 | .120 | .392 |
| Depression (RCADS) | 20.8 (6.70) | 18.9 (6.21) | *t* = 1.38 | .170 | .306 |
| Irritability (ARI-C) | 9.64 (4.27) | 7.36 (3.98) | *U* = 684.5 | .011 | .531 |
| Complex PTSD diagnosis* | 15 (51.7%) | 41 (58.6%) | *χ^2^* = 0.391 | .532 | .117 |
| Social support (MSPSS) | 57.4 (12.9) | 57.9 (13.0) | *t* = 0.185 | .854 | .041 |

**Note:** Significant results depicted in bold. A Bonferroni correction was applied for the three primary analyses and a Holm-Bonferroni correction was applied for the secondary analysis.
CPTCI – Post-Traumatic Cognitions Inventory, Child version; CRIES-8 = Child Revised Impact of Events Scale; TMQQ = Trauma Memory Quality Questionnaire; RCADS = Revised Child Anxiety and Depression Scale; CSBS = Child Safety Behaviour Scale; SDQ = Strengths and Difficulties Questionnaire; ARI-C = Affective Reactivity Index – Child version; MSPSS = Multidimensional Scale of Perceived Social Support.

*Categorical variables so frequencies rather than means are reported.

***Table S3: Logistic regression modelling of voice hearing, controlling for dissociation***

| Model number | Predictor variable | Model Nagelkerke R^2^ | Model  χ^2^ (df = 2) | Predictor variable Wald statistic | Predictor variable  Odds ratio |
| --- | --- | --- | --- | --- | --- |
| 1 | Negative cognitions (CPTCI) | .066 | 5.92 (*p* = .052) | 2.66 (*p* = .103) | 1.02 |
|  | Dissociation |  |  | .182 (*p* = .670) | 1.04 |
| 2 | Memory quality (TMQQ) | .188 | 18.1 (*p* < .001) | **12.25 (*p* < .001)** | 1.18 |
|  | Dissociation |  |  | .003 (*p* = .960) | 1.00 |
| 3 | Panic disorder (RCADS) | .091 | 8.43 (*p* = .015) | **4.82 (*p* = .028)** | 1.07 |
|  | Dissociation |  |  | .248 (*p* = .618) | 1.05 |

***Table S4: Logistic regression modelling of voice hearing, controlling for dissociation (using sensitivity analysis groups with participants with voices indistinguishable from intrusions/flashbacks removed)***

| Model number | Predictor variable | Model Nagelkerke R^2^ | Model  χ^2^ (df = 2) | Predictor variable Wald statistic | Predictor variable  Odds ratio |
| --- | --- | --- | --- | --- | --- |
| 1 | Negative cognitions (CPTCI) | .047 | 4.01 (*p* = .134) | .938 (*p* = .333) | 1.02 |
|  | Dissociation |  |  | .664 (*p* = .415) | 1.09 |
| 2 | Memory quality (TMQQ) | .122 | 11.0 (*p* < .004) | **6.94 (*p* = .008)** | 1.13 |
|  | Dissociation |  |  | .269 (*p* = .604) | 1.05 |
| 3 | Panic disorder (RCADS) | .070 | 6.21 (*p* = .045) | 2.82 (*p* = .093) | 1.06 |
|  | Dissociation |  |  | .564 (*p* = .453) | 1.07 |
